# Meditation and neurofeedback: A systematic scoping review, synthesis, and future directions

**DOI:** 10.1162/IMAG.a.1284

**Published:** 2026-06-24

**Authors:** Hagar Tal, Winson F.Z. Yang, Matthew D. Sacchet

**Affiliations:** Meditation Research Program, Department of Psychiatry, Massachusetts General Hospital, Harvard Medical School, Boston, MA, United States; Faculty of Psychology and Neuroscience, Maastricht University, Maastricht, Netherlands; Athinoula A. Martinos Center for Biomedical Imaging, Department of Radiology, Massachusetts General Hospital, Harvard Medical School, Charlestown, MA, United States

**Keywords:** neurofeedback, meditation, advanced meditation, cognitive enhancement, meditative development

## Abstract

Neurofeedback (NF) has been proposed as a tool to support and expand access to meditation practice. Despite growing interest, no comprehensive review has examined neurofeedback for meditation-based interventions (NF-MED) across the full spectrum of practices. Here, we conducted a scoping systematic review according to PRISMA guidelines, mapping the NF-MED field across both clinical and non-clinical contexts, with three aims: (1) assess the current state of NF-MED studies, (2) evaluate how NF-MED meditative constructs and whether neural modulation shows evidence of transfer to behavioral or phenomenological outcomes, and (3) provide methodological recommendations for future work. Our analysis shows that the field is dominated by proof-of-concept studies and marked by substantial heterogeneity in design, implementation, and outcome measures, which hampers the assessment of efficacy and applicability of NF-MED. While NF-MED has been shown to consistently modulate neural activity, evidence for corresponding improvements in behavior, phenomenology, or transferable meditative skills remains limited. Additional research is thus essential for determining whether NF-MED can help practitioners overcome common meditative barriers, such as anxiety and self-doubt, and whether NF-MED can accelerate meditative development across the training spectrum from novice to advanced meditators.

## 1 Introduction

Mastery of meditation can lead to profound experiences including profound bliss, peace, self-transcendence, and insight, and in some contemplative traditions is described as progressing toward meditative endpoints (Sacchet et al., 2024; Sparby & Sacchet, 2025a). Despite growing popularity, with roughly 10% growth in participation of meditation and mindfulness programs in the United States alone between 2002 and 2022 (Davies et al., 2024), mechanisms supporting mediative development remain understudied. Millions now engage with meditation through digital apps, wherein practice occurs at even lower doses (Creswell & Goldberg, 2025) and adherence falls below 55% after the first 30 days (Baumel et al., 2019; Lam et al., 2023). Without adequate support or awareness of traditional meditation development maps, obstacles may be misinterpreted as failure rather than integral parts of meditative development (Sparby & Sacchet, 2025a, 2025b).

Neurotechnology has been proposed as a potential means of scaffolding meditative development (Abellaneda-Pérez et al., 2024; Brandmeyer & Delorme, 2020; Cain, Brandmeyer, et al., 2024; Cain, Simonian, et al., 2024; Ganesan et al., 2025; Lord et al., 2024, 2025). Among neurotechnologies, neurofeedback is designed to enable participants to monitor and modulate their mind in real time using feedback derived from their own neural activity (Brandmeyer & Delorme, 2020; Brandmeyer et al., 2023; Sitaram et al., 2017; Weiskopf, 2012). This technology has demonstrated initial efficacy across clinical and cognitive enhancement contexts (Gruzelier, 2014a; Kohl et al., 2020; Niv, 2013; Paret et al., 2019), though methodological challenges remain (Gruzelier, 2014b; Thibault et al., 2018). In the context of meditation, neurofeedback may provide a mechanism to lower barriers for novices, sustain motivation through tangible feedback, and potentially facilitate access to advanced meditation.

Despite this promise, no comprehensive systematic review has yet examined the role of neurofeedback across meditation practices (NF-MED). Prior reviews have been non-systematic (J. C. C. Chen & Ziegler, 2025), or focused on clinical outcomes such as anxiety and stress reduction, without addressing meditative skill progression or depth (Treves et al., 2024). As a result, the field currently lacks an integrative synthesis that evaluates how NF-MED paradigms are designed, implemented, and evaluated, and that identifies methodological bottlenecks limiting causal inference and reproducibility (Ehmann et al., 2025). We address these gaps in the present systematic scoping review by systematically mapping the literature of NF-MED applied to meditation across both clinical and non-clinical contexts. Our goals are threefold: (1) to assess the current state of NF-MED studies, (2) to assess the extent to which neural modulation is accompanied by evidence of transfer to behavioral or phenomenological outcomes relevant to meditation training, and (3) to provide methodological and conceptual recommendations for future NF-MED research. In doing so, this review extends beyond prior work by integrating findings across modalities (electroencephalography [EEG], functional magnetic resonance imaging [fMRI], and functional near-infrared spectroscopy [fNIRS]), and neural and phenomenological targets, and study designs. We also outline a future-oriented framework for designing NF-MED studies capable of testing developmental claims, including in the longer term, meditative development and advanced meditation, flourishing, and meditative endpoints, under stronger evidential standards.

This review is structured as follows. In Section 2, we outline our search strategy and detail the process of data extraction and study selection in accordance with PRISMA guidelines. Section 3 summarizes general trends across the literature and provides an in-depth analysis of meditation protocols, neurofeedback modalities, feedback types, and reported neural, behavioral, and phenomenological outcomes. Section 4 situates these findings in the broader landscape of contemplative science, evaluates the potential and conditions under which neurofeedback may support meditative development, and outlines concrete methodological avenues to guide future work in this emerging research domain.

## 2 Methods

### 2.1 Review type

We conducted a systematic scoping review in accordance with the PRISMA-ScR guidelines to map the existing literature on concurrent NF-MED. This approach was selected given the heterogeneity of study designs, populations, and outcome measures, which precludes formal meta-analysis or conventional risk of bias synthesis.

### 2.2 Inclusion and exclusion criteria

We included empirical studies that employed NF-MED in both non-clinical and clinical settings. No restrictions were placed on NF-MED modality (e.g., EEG-, fMRI-, fNIRS-based), and the definition of meditation was kept broad to encompass a wide spectrum of practices. For this review, meditation was operationally defined as intentional, sustained, and open attention-oriented inward, rather than toward an external sensory stimulus. However, it is important to note that current definitions of meditation fail to integrate phenomenology, context, and purpose, which prevents a unified scientific understanding of meditation across its full developmental and cultural range. Defining meditation in terms of intentional, context-sensitive mental activity with identifiable phenomenological features would enable greater precision in linking specific practices to their underlying mechanisms, developmental trajectories, and outcomes (Sparby, 2022). Such an approach allows for differentiation between attentional, affective, and insight-oriented techniques, while preserving the traditional understanding of meditation as a transformative training of consciousness rather than a mere relaxation or wellness practice. This broader yet principled definition provides the conceptual foundation for evaluating how NF-MED may interact with distinct meditative processes and stages of development.

Exclusion criteria included non-neural biofeedback (e.g., heart rate variability, skin conductance), lack of concurrent NF-MED, literature reviews, theses, and dissertations. Additional exclusion criteria were the use of a commercial neurofeedback device, as they did not provide sufficient methodological transparency, such as those using proprietary algorithms without disclosure of signal processing pipelines, target features, or electrode configurations. Articles published in languages other than English were also excluded.

**Fig. 1.**
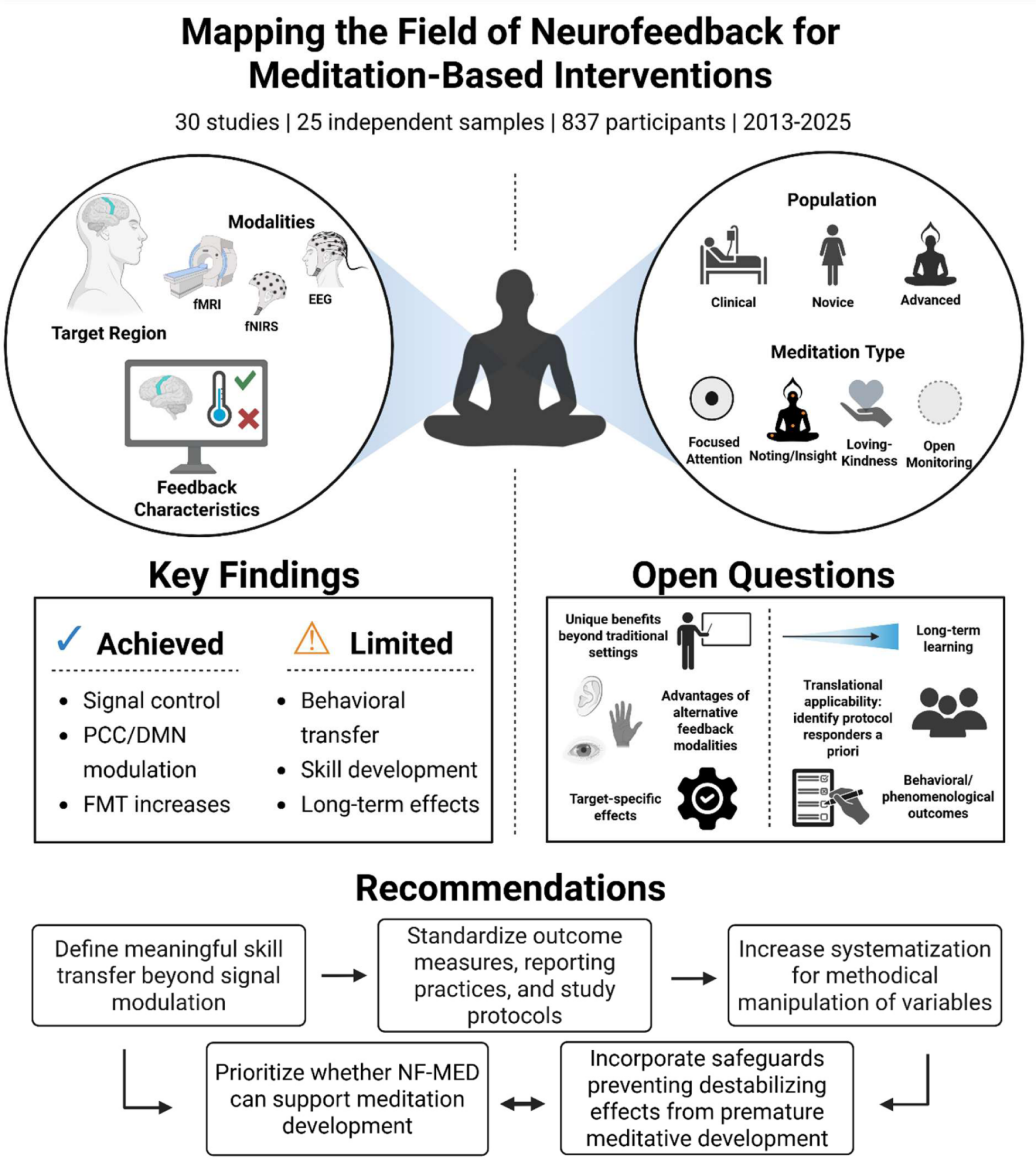
Mapping the Field of Neurofeedback in Meditation-Based Interventions: Study Characteristics, Key Findings, and Research Priorities. This figure provides a visual overview of the systematic scoping review examining neurofeedback-assisted meditation (NF-MED) interventions. Top center summarizes the scope of the review, including the total number of studies (30), independent samples (25), and participants (837) spanning 2013–2025. Top left panel summarizes key study components: neuroimaging modalities (electroencephalography [EEG], functional magnetic resonance imaging [fMRI], functional near-infrared spectroscopy [fNIRS]), neural target regions (e.g., posterior cingulate cortex [PCC], default mode network [DMN], or frontal midline theta [FMT]), and feedback characteristics (visual, auditory, multimodal presentation). Top right panel displays the diversity of study populations (clinical, novice, advanced meditators) and meditation types examined (focused attention [FA], open monitoring [OM], loving-kindness [LK], noting/insight practices). Middle left panel presents key findings organized into achieved outcomes (signal control, PCC modulation, frontal midline theta [FMT] increases) versus limited outcomes (behavioral transfer, skill development, long-term effects). Middle right panel outlines open questions requiring further investigation, including the optimal balance between standardization and target-specific approaches, potential advantages of NF-MED over traditional teacher-guided instruction, and the role of translational phenomenology in bridging neural and experiential outcomes. Bottom panel summarizes core methodological recommendations: defining transfer beyond signal modulation, standardizing outcome measures and reporting practices, rigorously manipulating and controlling methodological variables (sample size, control conditions, follow-up assessments), prioritizing research on whether NF-MED supports meditation development, and incorporating ethical safeguards against premature meditative advancement. Figure created with BioRender.com. NF-MED, neurofeedback-assisted meditation; EEG, electroencephalography; fMRI, functional magnetic resonance imaging; fNIRS, functional near-infrared spectroscopy; PCC, posterior cingulate cortex; DMN, default mode network; FMT, frontal midline theta.

### 2.3 Systematic search

The main search on Scopus, PsycINFO, Web of Science, and PubMed was completed between October 25, 2024, and December 5, 2024. An additional search was completed between the 30th of April and the 6th of May 2025. All references were uploaded to Covidence, a web-based collaboration software platform that streamlines the production of systematic and other literature reviews. Search terms were “(neurofeedback OR neuro feedback OR neural feedback) AND (meditation OR mindfulness)”.

### 2.4 Study selection

Following automated duplicate removal in Covidence, two reviewers (H.T., and a research assistant) independently screened all titles and abstracts against the eligibility criteria. At this stage, studies were excluded if they used commercial neurofeedback devices, met other exclusion criteria, or were clearly irrelevant. Examples of excluded categories include review articles, protocol papers, and conference abstracts without accessible full texts. Full-text screening was conducted for the remaining articles to confirm eligibility, including verification that neurofeedback and meditation were administered concurrently. Discrepancies at either stage were resolved through discussion, with a third reviewer (WY) adjudicating as needed.

### 2.5 Data extraction

Studies were grouped according to intervention (type of meditation) and population (clinical/non-clinical, long-term/novices). The studies were coded for sample information (e.g., size and description), control group and condition, intervention type, NF-MED information, neural and behavioral outcomes, and limitations. Intervention type included the type of meditation and meditation instructions, while NF-MED information encompassed both NF-MED modality (e.g., fMRI, brain region targeted) and feedback information such as feedback description or session duration, and the authors’ hypotheses and expected results. Neural and behavioral outcomes were coded for region and connectivity results, meditation and affect scales, clinical symptom improvement, post-neurofeedback task scores, and phenomenological reporting. These are presented in Tables 1 to 4.

**Table 1.** Technical setup for neurofeedback in meditation-based interventions (NF-MED).

		Modality		Feedback characteristics		
Citation	Design	Method	Target	Modality	Feedback cue	NF duration (runs x min, number of sessions)
**Long-Term Meditators**						
Garrison, Scheinost, et al., 2013	Neurophenomenology	fMRI	PCC	Visual	Direction and color	Exp. 1A&B: 6 × (30 s baseline + 3 min FA) + 1 × (30 s baseline + 3 min FA). Exp. 2: 13 × (30 s baseline + 1 min FA)
Garrison, Santoyo, et al., 2013	Neurophenomenology, feasibility study	fMRI	PCC	Visual	Direction and color	1 x 30 s baseline + 1 x 1 min NF (4 conditions, N runs each), 1 session. Meditation with offline feedback: 4 runs; Meditation on a graph with offline feedback: 3 runs; Meditation with real time feedback: 3 runs; Volitional manipulation of the feedback graph: 6 runs
Hinterberger and Fürnrohr (2016)	User study; within-subject crossover.	EEG	CPz; whole range from ultra-low potentials to gamma and wide range frequencies.	Multimodal (visual + auditory)	Light color, pitch, loudness	3 sessions x 20 min (2 conditions per session)
Prestel et al. (2019)	Feasibility	EEG	32 channels, EEG independent components (weighted sum of all electrodes) 2 electrodes (P7 & FC2) excluded due to noise; FMT	Visual	Brightness, size	5 x 5 min, 8 sessions, 2 weeks
van Lutterveld et al. (2017)	Double-blind, randomized	EEG	Gamma activity (40–57 Hz) from PCC (beamformer localization)	Visual	Direction (left–right)	1 x 30 s baseline, 4 x 1.5 min meditation with offline NF, 3 x 1.5 min rtNF meditation, 2 x 7 min “free-play” rtNF (participant-chosen practice), 3 x 1.5 min volitional manipulation of rtNF (“draw on previous experience”), 3 x 1.5 min volitional manipulation of rtNF in opposite direction from effortless awareness (“draw on previous experience”); 2 x 4 min meditation without feedback, 1 session
**Novice Meditators**						
Arpaia et al. (2022)	Feasibility	EEG	FCz, CPz; midline beta power	Visual	Color	22 x 37 s (2 conditions), 6 sessions (3 per condition)
Brandmeyer and Delorme (2020)	Double-blind, active-controlled, randomized	EEG	Fpz, FZ, F7, F8, Cz, P7, P8, Oz; FMT	Visual	Color	6 x 5 min, 8 sessions, 2 weeks.
Cosgrove et al. (2024)	Feasibility, single-arm	fMRI	PCC seed, DMN regions and regions of the SN	Visual	Color	1 x 6.56 min “observation”, 3 x 6.56 min blocked-design NF (66 s rest, 20 s describe, 70 s focus-on-breath, 30 s rest), 1 x 6.56 min TRS, 1 session
Fujii et al. (2019)	Feasibility	fNIRS		Auditory	Not described	10 min, 1 session
Ganesan et al. (2025)	Single-blind, RCT	fMRI	Bilateral ventral PCC compared with salience network and CEN for within-group analyses	Visual	Direction	1 x 2.5 min baseline, 3 x 10 min blocked-design NF (51 s rest, 26 s FA, 2.5 min TRS), 2 sessions
Kim et al. (2019)	Double-blind, RCT	fMRI	SN, DMN, CEN	Visual	Direction	2 x 7 min NF, 1 x 7 min TRS, 1 session
Kirlic et al. (2022)	Feasibility, single-arm	fMRI	PCC seed, DMN regions and regions of the SN	Visual	Color	5 x 6.56 min task-based (OBS, NF-1, NF-2, NF-3, and TRS), 8 runs
Kosunen et al. (2016)	System design, user study	EEG	F3, F4, C3, C4, P3, P4; average alpha and theta bands	Visual	Opacity, floating effect, star particle effects	1 x 5 min baseline, 6 x 10 min NF/VR conditions, 1 session
Lawrence et al. (2014)	Controlled	fMRI	Subject-specific raINS	Visual	Direction, color	4 x 5.6 min NF (5 × 30 s baseline/increase blocks), 2 x 5 min “affective probe” runs (9 × 22 s increase/baseline feedback blocks + 8 s IAPS images).
Salminen et al. (2018)	Controlled	EEG	F3, F4, C3, C4, P3, P4; frontal alpha asymmetry	Visual	Pulsation, illumination, color	1 x 2 min baseline, 1 x 6.5 min NF (8 conditions), 1 session
Salminen et al. (2022)	System design, controlled	EEG	F3, F4, C3, C4, P3, P4; frontal asymmetry alpha band. Synchrony between participants was calculated as asymmetry values being within the same percentage of the individual range	Visual	Pulsation, illumination, color	1 x 2 min baseline, 1 x 6 min NF (8 conditions), 1 session
Salminen et al. (2024)	Controlled	EEG	Bilateral frontal theta (average of F3 and F4) and bilateral frontal alpha (average of F3, F4, C3, C4, P3, and P4)	Visual	Direction, opacity	1 x 5 min baseline, 6 x 10 min randomized conditions, 1 session
Saxena et al. (2023)	Pre-registered, single-arm, feasibility	fMRI	Subject-specific TPJ and ToM network	Visual	Number increase/decrease and size of smile	3 x training task (20 s fixation, 6 x 30 s NF (3 up- and 3 downregulation), 4 s feedback)
Soriano et al. (2024)	Pseudorandomized controlled, feasibility	EEG	22 electrodes; absolute alpha power averaged over all electrodes	Auditory	Loudness	2 x 3 min baseline, 6 x 2 min NF, 4 runs, 1 session
**Clinical Populations**						
Bauer et al. (2020)	Non-blind, randomized, controlled, feasibility	fMRI	DMN & CEN	Visual	Direction, size, and color	6 x 2.5 min experimental condition; 6 x 2.5 min control (sham), 1 session
Bauer et al. (2025)	Single-blind, RCT	fMRI	Subject-specific STG	Visual	Color	2.5 min baseline, 4 x 2.5 min NF-blocked design (16 s task, 4 s rest with feedback presentation); 1 x 2.5 min TRS, 1 session
C. Chen, Yu, et al. (2021)	Controlled	EEG	FP1, Fp4, F3, F4, F7, F8; bilateral alpha power	Visual	Color	8 min, 1 session
C. Chen, Xiao, et al. (2021) [Bibr IMAG.a.1284-b18]	Controlled	EEG	Fp1, Fp2, F7, F3, F4, F8, T7, C3, C4, T8, P7, P3, Pz, P4, P8, O1, O2, Fz, Cz; bilateral alpha power	Visual	Color	8 min, 1 session
Morfini et al. (2024)	Feasibility, single-blind RCT	fMRI	Subject-specific STG.	Visual	Direction, static color	4 x 3 min (20 s NF, 30 s rest), 1 session
Percik et al. (2019)	Feasibility, single-arm	NIR HEG	Fp1 and Fp2; the device shines light of wavelengths 660 nm and 850 nm through emission probes that were placed 3 cm lateral from the detection probes along the T3-Fpz-T4. The targeted regions were the superior orbitofrontal cortex and anterior cingulate cortex.	Visual	Acceleration	1 x 30 min, 10 sessions, 5 weeks
Yu et al. (2025)	Single-blind RCT	fMRI	Subject-specific STG.	Visual	Color, direction	4 x 2.5 min, 1 session
Zhang et al. (2023)	Feasibility, single-arm	fMRI	MN & CEN	Visual	Direction and size	5 x 2.5 min, 1 session
Zhang, Tusuzian, et al. (2025)	RCT	fMRI	Subject-specific DMN	Visual	Color	1 x baseline run, 4 x 2.13 min blocked design NF (4 x 16 s). Baseline length not reported
Zhang, Bauer, et al. (2025)	RCT	fMRI	PCC	Visual	Direction	5 x 6.56 min (3 NF, Obs, Trs), 2 x 6 min (Pre/Post resting state), 1 session

Systematic review of studies using neurofeedback to support meditation (NF-MED). Studies are organized by target population: long-term meditators are listed first, followed by meditation novices, and finally clinical populations. The Design column presents the type of study. The Modality column indicates whether the study employed neuroimaging, electrophysiology, or functional infrared spectroscopy, and what were the NF targets (electrodes if EEG or brain area if fMRI/fNIRS). Electrode locations are on 10–20 system unless otherwise specified. The Feedback Characteristics column details feedback specifications including whether feedback presentation was visual or auditory, feedback properties, and the study’s duration, including feedback dosage. Only total study duration is indicated when other information was unavailable.

Feasibility study = a study that assesses the practicality of a given method, often with small sample sizes; system design/user study = a systematic investigation of users to understand their needs, behaviors, and preferences when interacting with a product, service, or system; EEG = electroencephalography; fMRI = functional magnetic resonance imaging; fNIRS = functional infrared spectroscopy; NIR-HEG = near-infrared hemoencephalography; RCT = randomized controlled trial; FA = focused attention; OM = open monitoring; LK = loving-kindness; FMT = frontal medial theta; VR = virtual reality; MC = motor cortex; SMC = supplementary motor cortex; PCC = posterior cingulate cortex; rINS = right insula; FC = functional connectivity; PCC = posterior cingulate cortex; mPFC = medial prefrontal cortex; ACC = anterior cingulate cortex; RTPJ = right temporoparietal junction; DLPFC = dorsolateral prefrontal cortex; DMN = default mode network; SCR = skin conductance response; PDA = positive diametric activity; MEDEQ = meditation depth questionnaire; TRS = transfer run; OBS = observation; BCT = breath counting task. HC = healthy controls; ELA = early life adversity; AH = auditory hallucinations; RT = reaction time.

### 2.6 Bias and quality coding

No automation tools were used beyond Covidence’s duplicate rejection software. H.T. and a research assistant independently extracted the data from all studies, and a second reviewer (H.T. or W.F.Z.Y.) confirmed the accuracy of the extracted data. The selection criteria were kept stable throughout the process, and discrepancies in coding were discussed among the three coding authors and resolved before completion of the data extraction. Given the conceptual and methodological heterogeneity of NF-MED research, and the early developmental stage of the field, a formal risk-of-bias assessment or graded evidence synthesis was considered unlikely to yield meaningful comparative inferences and, therefore, fell outside the scope of this review. Furthermore, no effect measures were extracted. Instead, we systematically extracted the study limitations (e.g., small sample sizes, lack of control groups, short-term follow-up) and discussed them in the Discussion to provide a narrative appraisal of methodological quality.

## 3 Results

**Fig. 2.**
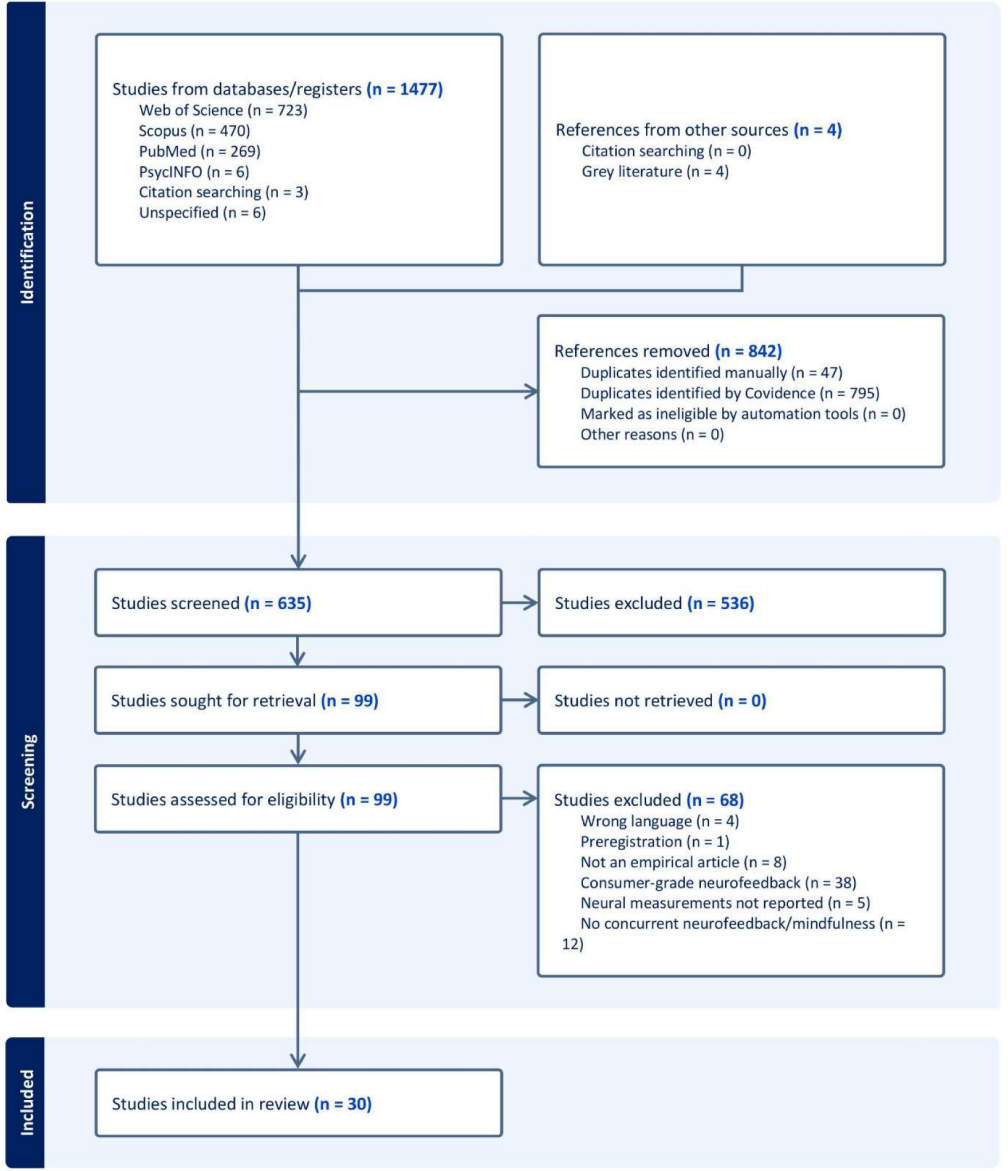
PRISMA-ScR flow diagram illustrating the selection process of NF-MED studies for inclusion in the systematic scoping review. The diagram outlines the number of records identified through database searching and other sources, the number of records after duplicates were removed, the number of records screened, full-text articles assessed for eligibility, and the final number of studies included in the synthesis.

The search yielded 1,473 records across 4 databases, with an additional 4 studies from gray literature. After removing duplicates and excluding based on title and abstract, full texts were reviewed for the remaining 99 studies. The final sample included 30 studies with 25 independent samples representing 837 participants. A PRISMA flow diagram is shown in Figure 2. Two study protocols for randomized controlled trials (Bloom et al., 2023; Weiss et al., 2020) that adhered to the inclusion criteria were excluded due to the lack of reported results.

### 3.1 Brief summary

The resulting sample spanned just over a decade, beginning with Garrison, Santoyo, et al. (2013). A total of 11 studies featured clinical populations, 4 of which included the same sample and are randomized controlled trials (RCTs) (Bauer et al., 2025; Morfini et al., 2024; Zhang, Bauer, et al., 2025; Zhang, Tusuzian, et al., 2025), in addition to Yu et al. (2025). Three additional studies with a non-clinical novice population were RCTs (Brandmeyer & Delorme, 2020; Ganesan et al., 2025; Kim et al., 2019). Follow-up was carried out on five independent samples (Bauer et al., 2025; Ganesan et al., 2025; Kirlic et al., 2022; Morfini et al., 2024; Percik et al., 2019; Yu et al., 2025; Zhang, Tusuzian, et al., 2025), and ranged from 1 week to 3 months. None of the studies involved a longitudinal design. Most studies (*N* = 23) involved fewer than 40 participants (median = 28), and a nearly equal number of studies used EEG and fMRI, while 2 studies applied fNIRS (Fujii et al., 2019; Percik et al., 2019). Finally, seven studies reported the CRED-nf checklist (Cosgrove et al., 2024; Ganesan et al., 2025; Kirlic et al., 2021, 2022; Morfini et al., 2024; Saxena et al., 2023; Yu et al., 2025).

### 3.2 Meditation expertise, meditation strategies, and instructions

Meditation expertise was generally assessed by estimates of lifetime meditation hours. Novices were defined as those with no meditation experience (Arpaia et al., 2022; Soriano, de Oca, et al., 2024), fewer than 20 (Van Lutterveld et al., 2024) or less than 500 lifetime but irregular meditation hours (Ganesan et al., 2025), or were not further specified. Long-term meditators reported between 100 and 10,567 h of practice, spanning 5 to 18 practice years on average. Except for Garrison, Scheinost, et al. (2013, experiment 1a), who specifically selected meditators practicing within the Theravada Buddhist tradition, all other studies collapsed across individuals who practiced across different contemplative traditions. NF-MED strategies included focused attention (FA) meditation (*N* = 14 studies) and noting/insight meditation (*N* = 11). The remaining eight studies included loving-kindness (LK), open monitoring (OM), or allowed participants to define their own strategies with post hoc categorization or description. Two studies (one sample) lacked sufficient detail for categorization. Study designs did not often involve comparison between meditation strategies, and when they did, both significant (Salminen et al., 2024) and non-significant (Kosunen et al., 2016) results were found. Crucially, only nine studies reported verifying that participants implemented the required meditation strategy (Brandmeyer & Delorme, 2020; Ganesan et al., 2025; Garrison, Scheinost, et al., 2013; Kirlic et al., 2022; Lawrence et al., 2014; Prestel et al., 2019; Saxena et al., 2023; Van Lutterveld et al., 2017; Zhang et al., 2023).

Concentration practices mainly included FA meditation (Lutz et al., 2008). While differing in the meditation object, experiential tone, and intensity (Sparby & Sacchet, 2022), these practices typically involved a narrow attentional aperture and, especially in novices, repeated redirection of focus toward the chosen object when the mind wanders (Lutz et al., 2015). Breath was the most common object of focus, but other objects included general bodily sensations (Brandmeyer & Delorme, 2020), the crown of the head (Soriano, de Oca, et al., 2024), a visual point (Kosunen et al., 2016), or the neurofeedback display graph itself (Garrison, Santoyo, et al., 2013; Garrison, Scheinost, et al., 2013).

Mahasi-style noting meditation (*N* = 9), which involves non-judgmental labeling of present-moment experiences as they arise and pass, while avoiding judgment, was used mostly with clinical populations except for two studies (Arpaia et al., 2022; Van Lutterveld et al., 2017). Only one study (Van Lutterveld et al., 2024) reported explicit instructions. Goenka-style noting, particularly body scanning, in which the noting focuses on bodily sensations, was studied in another three reports (Kosunen et al., 2016; Lawrence et al., 2014; Salminen et al., 2024).

LK meditation (*N* = 2), which emphasizes cultivating feelings of compassion and goodwill toward self and others (Burbea, 2014; Hofmann et al., 2011), was implemented in a virtual-reality context, where dyads directed compassion toward avatars representing each other to enhance affective interdependence and empathy (Salminen et al., 2018, 2022). However, precise meditation instructions were not reported.

Only Prestel et al. (2019) used OM. Three studies (C. Chen, Xiao, et al., 2021; C. Chen, Yu, et al., 2021; Percik et al., 2019) did not provide enough instruction details, though two seemingly used pre-recorded mindfulness instructions without further specification. These recordings have not been made available, and the cited references for the same recordings appear to be incorrect.

Finally, four studies, mainly involving long-term meditators, allowed participants to define their own meditation strategies. Saxena et al. (2023) encouraged participants to choose their own strategy and to report after each neurofeedback session, without providing them with any meditation instructions. Van Lutterveld et al. (2017), Garrison, Scheinost, et al. (2013) and Garrison, Santoyo, et al. (2013) included a “free-play” run, where participants were encouraged to use whichever strategy they found most effective for modulation of the neurofeedback signal. Similarly, Prestel et al. (2019) allowed participants to explore strategies beyond FA and OM, including visualization, attempting to impose one’s will on the feedback graph, noting, and attempting to evoke feelings of well-being.

In sum, these studies highlight substantial heterogeneity in meditation experience and lack of standardization in instructed practices. These aspects underscore the need for pre-specified instruction protocols that ensure comparability within any given meditation practice—for instance, explicitly differentiating between target attentional qualities during FA, instructed activity during loving-kindness, or tradition and lineage when recruiting advanced meditators, ultimately capturing the first-person phenomenology and mental activities that define each practice (Lutz et al., 2015; Sparby & Sacchet, 2022). Alongside enhanced verification of adherence to intended techniques through post-session questionnaires and phenomenological interviews, such modifications would minimize variability and improve specificity and clarity in implemented meditation strategies. Greater specificity enables two complementary advances: first, it allows meaningful comparison across studies to identify the active components of different meditation–neurofeedback pairings; second, it provides the foundation for eventual personalization, wherein training regimens can be tailored to individual participants based on their neural response profiles and experiential capacities. Standardization of reporting thus serves diversity of application.

### 3.3 Control groups and conditions

Most studies included a within-group control condition, while fewer had a between-group comparison, and two (Prestel et al., 2019; Zhang et al., 2023) had no control group of either type. Within-group control conditions included sham feedback (e.g., non-target region, typically motor cortex, yoked/offline; *N* = 4), meditation-only (*N* = 4), and bidirectional modulation (*N* = 5; see Tables 2–4). Remaining studies compared a resting or active baseline to post-NF-MED activity. Between-group controls were largely constituted by novices or healthy controls receiving active (*N* = 6) or sham NF-MED (*N* = 8). Of these studies, only two compared subjects on behavioral outcome, rather than on brain activation. Finally, several studies compared the efficacy of physiological feedback against neural feedback.

**Table 2.** Characteristics and outcomes of neurofeedback-assisted meditation studies in long-term meditator populations.

	Experimental group		Control group		Findings	
Citation	Sample	Meditation	Between-group	Within-group	Neural	Behavioral and phenomenological
Garrison, Scheinost, et al. (2013)	Exp. 1: 22 adult meditators (Theravada). Total = 9,249 ± 1,449 practice hours (12.0 ± 1.6 years). Exp 2: 10 adult meditators (different traditions). Total = 10,567 ± 4,276 practice hours (18.4 ± 4.9 years).	FA applied to different objects	Exp. 1a: 22 novices, case-matched. Exp 1B: 11 novices drawn from 1A. Exp 2: N/A	Sham feedback; bidirectional modulation; active baseline.	Meditators vs non-meditators → PCC deactivation but uncorrelated with meditation experience; no correlation between meditation hours and signal change.	Both groups → significant association between subjective experience and feedback graph (runs 1–4); could discriminate PCC vs posterior parietal feedback; all participants → coupling between focused attention and ↓ PCC activity by end of discovery step 1; meditators → significant correspondence between feedback and FA experience; all participants reported following instructions;
Garrison, Santoyo, et al. (2013)	10 (3 female) meditators (different traditions). Total = 10,567 ± 4,276 practice hours (18.4 ± 4.9 years; daily + retreats).	FA applied to experience	N/A	Active baseline; sham feedback; bidirectional modulation.	Undistracted awareness and effortless doing → PCC deactivation; self-related thought, control, or distracted awareness → PCC activation.	None reported.
Hinterberger and Fürnrohr (2016)	36 (21 female) adults. 26 meditators (different traditions). Mean = 8.2 years, 3.1 times/week.	Mindfulness	10 non-meditators, post hoc comparison on subjective feedback ratings only	Counterbalanced crossover design; active control mindfulness and body-scan; biofeedback; offline yoked-sham feedback; different feedback sequence.	All Sensorium conditions → ↓ power across all frequencies, especially with body scanning.	Long-term meditators benefited from all six conditions; non-meditators benefited more from Sensorium; well-being and perceptual improvements observed in all conditions; sensorium rated more “extraordinary”; no other NF vs non-NF differences found.
van Lutterveld et al. (2017)	16 adult novices (no meditation in previous year and >20 lifetime meditation hours); 16 adult meditators (30 min/day ≥ 5 days/week ≥ 5 years; various traditions). Median lifetime hours = 6,164	Noting	Novice vs meditators compared on behavioral measures	Offline NF and double-blind randomized bidirectional modulation	Effortless awareness achieved by novices and long-term meditators → PCC deactivation in both, no above-chance volitional increase achieved; no group differences observed; significant confounds.	Subjective experience associated with activity direction, with high confidence and no group differences.
Prestel et al. (2019)	6 (2 female) meditators (different traditions). Mean = 100 h (10 min–40 min/day).	FA applied to breath, OM	N/A	N/A	High FMT power linked to well-being and effortless confidence; signal evaluation had negative effects; FMT power dropped only after giving up; subjective performance appraisals did not match objective results, influenced by selective perception.	All participants developed personal strategies beyond FA/OM; effective strategies involved relaxed concentration without object, acceptance, and positive feelings.

This and subsequent tables display the systematic review of studies examining neurofeedback-assisted meditation (NF-MED) interventions in long-term, novice, and clinical meditator populations, respectively. The Experimental Group columns describe the sample characteristics and type of meditation practice. Control Group columns indicate the comparison conditions used, including between-group controls (separate control participants) and within-group controls (repeated measures within the same participants). The Findings columns summarize primary outcomes, with Neural findings reporting neuroimaging or electrophysiological results, and Behavioral findings reporting phenomenological, psychological, or cognitive outcomes. See Table 1 for abbreviations.

**Table 3.** Characteristics and outcomes of neurofeedback-assisted meditation studies in novice meditator populations.

	Experimental group		Control group		Findings	
Citation	Sample	Meditation	Between-group	Within-group	Neural	Behavioral
Kosunen et al. (2016)	43 (26 female) university students.	FA, Body Scan	N/A	Meditation without NF/VR; meditation type.	Not reported	VR-NF → ↑ meditative experience (MEDEQ, ITC-SP); controls showed ↑ negative affect and ↓ relaxation; no difference between meditation types.
Lawrence et al. (2014)	24 (12 female) adults.	Body Scan	8 participants received sham feedback from the parahippocampal area.	Baseline vs increase blocks; pre–post affective probe runs.	Successful regulation of rINS during feedback; subjective experience was not reflected; other areas were also modulated.	No group differences in affect ratings; no SCR response in nine participants; sham group exerted more effort and exploration.
Salminen et al. (2018)	44 (43 female) adults.	LK	N/A	Dyadic and solo meditation without NF; biofeedback (respiration).	None reported.	NF vs control and respiratory feedback → ↑ affective interdependence.
Kim et al. (2019)	60 male university students.	FA applied to breath	30 participants received yoked sham NF.	N/A	No significant FC enhancement between groups during training or TRS.	Both groups → ↑ mindfulness post-NF despite differing mood trajectories.
Brandmeyer and Delorme (2020)	24 (12 female) adults.	FA applied to various objects	12 (6 women) participants received yoked sham NF.	Baseline activity.	Significant group/session differences in FMT and EEG spectra; NF → ↑ gamma and FMT power vs sham during N-back task.	NF group responded faster on correct N-back trials; no significant differences pre/post on sustained attention and conflict detection tasks.
Kirlic et al. (2022)	34 (14 female). Adolescents (mean age = 15 years).	FA applied to breath	N/A	Baseline and active baseline (“Describe” condition).	PCC deactivation during NF-MED, and correlated with activity in mPFC, dACC, insula, hippocampus, and amygdala.	No group difference in ease of focus nor other measures of stress or affect.
Arpaia et al. (2022)	4 (1 female) adults.	Noting	N/A	Cognitive reappraisal task	Three subjects exhibited reduced high-beta power during NF condition.	Emotional acceptance rated more difficult than cognitive reappraisal; PSD trend visually similar in both conditions.
Salminen et al. (2022)	72 adults (44 female).	LK	N/A	No bio-signal modulation of VR; biofeedback (respiration).	Frontal asymmetry correlated with empathy ratings; no actor/partner effect.	NF increased self-reported empathy, especially in dyadic condition.
Saxena et al. (2023)	16 (9 female) adults.	Self-defined	N/A	Bidirectional modulation.	↑ activation during upregulation; smaller TRS effects in all regions except mPFC and RTPJ. No session effects or condition x session interaction.	TRS strength → improved hinting task performance; Upregulation → social, affiliation, drive explained variance in all ROIs except PC, VMPFC; Downregulation → perceptual processes, seeing features explained variance in DMPFC, MMPFC, RSTS, VMPFC.
Ganesan et al. (2025)	40 adults (27 female). Lifetime experience < 500 h	FA applied to breath	20 participants received yoked sham feedback.	Baseline.	↑ PCC–DLPFC decoupling in final real NF-MED training in experimental group → ↓ emotional distress; no group differences in PCC deactivation or right DLPFC–PCC coupling during TRS.	NF → ↑ mindful awareness and ↓ distress; BCT performance ↓ baseline to follow-up; mindful awareness negatively correlated with BCT.
Cosgrove et al. (2024)	31 (14 female). Same sample as Kirlic et al. (2022).	FA applied to breath	N/A	Baseline and active baseline (“Describe” condition).	↑ PCC–hippocampus/amygdala connectivity; no ↓ PCC–DMN connectivity, no ↑ PCC-SN connectivity.	None significant.
Soriano et al. (2024)	31 (25 female) adults.	FA applied to crown of the head	N/A	Alpha downregulation.	Successful alpha power modulation over temporal and occipital regions. No TRS.	No significant change in self-reported measures of tiredness, pleasantness, calmness, or focus.
Salminen et al. (2024)	43 (26 female) university students. Same sample as Kosunen et al. (2016).	FA, Body Scan	N/A	No NF (VR/no VR).	NF → ↑ frontal theta (FA) and gamma (body-scan); no change in alpha.	NF → ↑ sense of presence and MEDEQ scores; VR → ↓ reported hindrances.

See Table 1 for abbreviations.

**Table 4.** Characteristics and outcomes of neurofeedback-assisted meditation studies in clinical populations.

	Experimental group		Control group		Findings	
Author	Sample	Practice	Between-group	Within-group	Neural	Behavioral
Percik et al. (2019)	6 adult males. Overweight/mild obesity.	FA, Mindfulness	N/A	5 min of no-NF mindfulness.	Increased orbitofrontal activity and volume after NF.	Significant weight loss and reductions in emotional, external, and overeating at 6-month follow-up.
Bauer et al. (2020)	11 (1 female) adults. Schizophrenia/schizoaffective disorder.	Noting	N/A	Sham feedback from SMC at least 12 weeks post-real NF.	NF → ↓ PCC–mPFC FC and ↑ ACC–MPFC/rDLPFC anticorrelation; connectivity changes correlated with reductions in AH scores.	↑ PDA and ↓ AH scores 1 W post-NF; no changes post-SMC feedback.
C. Chen, Yu, et al. (2021)	34 adult anxiety patients.	Mindfulness	17 healthy controls.	Baseline pre- and post-NF	None reported.	EEG classifier achieved 92.30 ± 1.31% accuracy, distinguishing anxiety levels.
C. Chen, Xiao, et al. (2021)	34 adult anxiety patients. Same sample as C. Chen, Yu, et al. (2021).	Mindfulness	17 healthy controls.	Baseline pre- and post-NF	Anxious patients: ↑ baseline power and widespread ↑ post-NF; HC: stronger alpha, theta, gamma modulation with mindfulness.	NF reduced anxiety in both groups, more prominently in anxious participants.
Zhang et al. (2023)	9 (6 female) adolescents. MDD/anxiety patients (self-reported).	Noting	N/A	N/A	↓ sgACC–mPFC connectivity correlated with mindfulness gains; FC change mediated mindfulness improvement.	Higher NF performance predicted ↑ state mindfulness (total, mind, body); ↓ sgACC–mPFC FC associated with greater mindfulness increase post NF-MED.
Morfini et al. (2024)	22 adults (4 female). Schizophrenia/schizoaffective disorder; frequent medication-resistant AHs.	Noting	11 participants received sham NF from MC.	Bidirectional modulation	7 significant clusters in self-reference network → ↑ activation in experimental group; NF > sham activation in mPFC, ACC, frontal cortex; baseline differences observed.	No significant association between neural change and hallucination severity.
Bauer et al. (2025)	25 adults. Schizophrenia/schizoaffective disorder	Noting	13 participants received sham NF from MC.	Bidirectional modulation	↓ ACC activation and ACC–rDLPFC connectivity in active vs sham; effect not replicated in sham-to-NF crossover.	Both groups showed decreased hallucination severity (PSYRATS-AH).
Zhang, Tusuzian, et al. (2025)	25 adults. Schizophrenia/schizoaffective disorder. Same as Bauer et al. (2025).	Noting	13 participants received sham NF from MC.	Bidirectional modulation	No group differences overall; greater symptom improvement linked to stronger DMN–PCC connectivity and DMN–DLPFC anticorrelation.	None reported.
Yu et al. (2025)	41 (17 female) adolescents (mean age = 15.04 years) suffering from ELA.	FA applied to breath	34 healthy controls (16 female). 20 adolescents with ELA (14 female) underwent no-NF MED.	N/A	NF → ↑ PCC downregulation in ELA (stronger modulation vs sham); HC > ELA in PCC deactivation; ELA-NF > ELA-sham in PCC-ACC connectivity.	Group × time: ELA-NF > HC in ↑ positive affect, ↓ stress at one-week follow-up; HC: ↑ mindfulness of body over time; ELA-NF: ↑ body mindfulness post only; mindfulness of the mind increased over time regardless of group.
Zhang, Bauer, et al. (2025)	25 adults. Schizophrenia/schizoaffective disorder. Same as Bauer et al. (2025).	Noting	13 participants received sham NF from MC.	N/A	Real-NF → ↓ MPFC–AC/lIFG connectivity; sham-NF → ↑ connectivity; AHs not associated with MPFC–PCC or MPFC–STG/MTG connectivity changes.	AHs significantly reduced post-NF for both groups.

See Table 1 for abbreviations.

### 3.4 Outcome measures

Measured outcomes spanned behavioral, phenomenological, and neural domains. Behavioral instruments included mindfulness (Yu et al., 2025), Independent Television Commission sense of presence (ITC-SOP) (Kosunen et al., 2016), imagery vividness (Saxena et al., 2023), affective and sensory awareness (Yu et al., 2025), empathy (Saxena et al., 2023), and mood (Kim et al., 2019). Clinical studies additionally used disorder-specific instruments (e.g., Psychotic Symptom Rating Scale – Auditory Hallucinations for auditory hallucinations in schizophrenia). Most studies administered these measures before and after the NF-MED protocol, though several also administered them between neurofeedback runs, and one study reported no baseline assessment (Zhang, Bauer, et al., 2025). Cognitive tasks (*N* = 4) assessed attention, working memory, and cognitive control (Brandmeyer & Delorme, 2020; Fujii et al., 2019; Salminen et al., 2024; Saxena et al., 2023), but were too heterogeneous for comparability across studies. Meditative state, depth, and intensity were rarely assessed (*N* = 5 studies included an assessment). The Meditation Depth Questionnaire was administered in only one sample (Kosunen et al., 2016; Salminen et al., 2024), and four others collected reports on phenomenology and strategies, linking them to NF-related neural outcomes (Garrison, Santoyo, et al., 2013; Garrison, Scheinost, et al., 2013; Prestel et al., 2019; Van Lutterveld et al., 2017). Neural outcomes included modulation (up- or downregulation) of specific brain regions or frequency bands, as well as changes in functional connectivity, synthesized in Section 3.6 *(Neural targets)* and in Tables 2–4.

### 3.5 Feedback modalities and presentation

Visual feedback was most common (thermometers or geometrical shapes; red–green or red–blue color schemes). Immersive virtual-reality (Kosunen et al., 2016; Salminen et al., 2018, 2022, 2024) or multimodal audio-visual environments (Hinterberger & Fürnrohr, 2016) were used in a few studies. Two studies used auditory-only feedback (Fujii et al., 2019; Soriano, de Oca, et al., 2024). Only two studies (Fujii et al., 2019; Soriano, de Oca, et al., 2024) reported NF-MED protocol was performed with eyes closed while one study (Van Lutterveld et al., 2017) allowed for participant choice. The remaining studies either explicitly reported eyes-open practice or did not specify. Protocol durations ranged from a single 10-min session (C. Chen, Xiao, et al., 2021; C. Chen, Yu, et al., 2021; Lawrence et al., 2014) to multi-session interventions across 2 to 5 weeks (Percik et al., 2019; Prestel et al., 2019).

### 3.6 Neural targets

Across fMRI and fNIRS studies, network targets frequently involved the default mode network (DMN) and its connectivity with the central executive network (CEN) and salience network (SN). A common goal was to downregulate these connections to reduce self-referential rumination (e.g., strengthening CEN-DMN anticorrelation) as CEN-DMN anticorrelation has been proposed as a marker of early meditative development (Cooper et al., 2022). The SN, which includes the insula, is linked to interoceptive processing (Craig, 2009; Seeley, 2019), and its modulation has been associated with increased state mindfulness. Specific regions were also frequently targeted to influence self-referential processing. The posterior cingulate cortex (PCC) was the most common target, with its downregulation or downregulation of its connectivity with other DMN hubs linked to decreased stress, negative affect, and self-focused thought. Clinical targets included STG (auditory hallucinations; Northoff, 2015; Whitfield-Gabrieli & Ford, 2012), and sgACC (rumination/affect) via medial prefrontal cortex to DMN coupling. One study (Saxena et al., 2023) investigated the temporoparietal junction within the theory of mind network to enhance pro-social attitudes through volitional control of this network.

EEG-based protocols spanned all major frequency bands, with alpha and theta being most commonly used. Alpha training has been used to increase empathy (Salminen et al., 2018, 2022), decrease anxiety (C. Chen, Xiao, et al., 2021; C. Chen, Yu, et al., 2021), and improve meditation effectiveness (Salminen et al., 2024; Soriano, de Oca, et al., 2024). Theta has been associated with focused attention and decreased mind-wandering (Brandmeyer & Delorme, 2020; Prestel et al., 2019) and anxiety (C. Chen, Xiao, et al., 2021). Beta activity has been linked to emotional regulation, specifically acceptance (Arpaia et al., 2022). Gamma was investigated in relation to equanimity (Van Lutterveld et al., 2017). Feedback electrodes varied, with some studies focusing on a subset, often the frontal or medial ones (e.g., frontal medial theta; FMT), while others used whole-head configurations. Except for one study (Van Lutterveld et al., 2017), which used an active electrode cap with its own denser layout and narrowband linear-constraint minimum-variance localization of the target area, all EEG sites are in the 10–20 system. Exact electrodes are reported in Table 1.

### 3.7 NF-MED in non-clinical cohorts

#### 3.7.1 Long-term meditators

PCC-targeted fMRI-neurofeedback linked effortless awareness and focused but relaxed concentration with PCC deactivation (Garrison, Santoyo, et al., 2013; Garrison, Scheinost, et al., 2013; Van Lutterveld et al., 2017). FMT increases showed associations with relaxed focus/acceptance (Prestel et al., 2019) while the opposite direction of modulation corresponded to effortful doing, judgment and attempts to actively control the signal in all studies. Nevertheless, it remains unclear whether these results apply to both novices and long-term meditators or only long-term meditators, given that Garrison, Scheinost, et al. (2013) found group differences but not van Lutterveld et al. (2017). User-experience studies favored immersive neurofeedback over meditation alone (Hinterberger & Fürnrohr, 2016). However, these studies were limited in their investigation of a single dimension of experience and lacked behavioral measures, control condition, or baseline recording. While no study included follow-ups, all noted short-term increases in well-being, motivation, or perceptual vividness (e.g., Hinterberger & Fürnrohr, 2016; Prestel et al., 2019). Taken together, these studies suggest that neurofeedback can reliably reflect and possibly enhance states of effortless awareness in long-term meditators, though evidence remains preliminary and mechanistically inconclusive due to small sample sizes, limited control conditions, and potential confounds arising from these limitations, including artifact contamination and open-eyed practice.

#### 3.7.2 Novices

Half of the included studies focused on non-clinical novices, either adults (Arpaia et al., 2022; Brandmeyer & Delorme, 2020; Fujii et al., 2019; Ganesan et al., 2025; Kim et al., 2019; Kosunen et al., 2016; Lawrence et al., 2014; Salminen et al., 2018, 2022, 2024; Saxena et al., 2023; Soriano, de Oca, et al., 2024) or adolescents (Cosgrove et al., 2024; Kirlic et al., 2021, 2022), with the latter reviewed separately due to distinct developmental and methodological considerations. These studies primarily aimed to assess whether neurofeedback enhances meditation-related outcomes (e.g., mindfulness, affect, empathy) and to evaluate the feasibility of modulating neural meditation markers using neurofeedback.

Across modalities, novices generally learned to modulate neural targets during training but did not sustain altered neural activity during transfer tasks (i.e., meditation runs without real-time feedback), and behavioral changes were inconsistent. In fMRI studies, Lawrence et al. (2014) found that participants were able to learn regulation of the right anterior insula in a single session, but other areas were also modulated, and arousal-related changes did not differ between groups or across time. Kim et al. (2019) observed increased SN–DMN connectivity, mediated by the CEN, which correlated with mindfulness scores, but task performance was not sustained during a subsequent transfer run. Saxena et al. (2023) reported robust activation of theory of mind regions during upregulation without whole-brain changes, with behavioral gains limited to a correlation between right temporoparietal junction activity and theory of mind task performance. Ganesan et al. (2025) found PCC-DLPFC negative coupling and mindfulness gains during a practice week without PCC-sham differences in modulation.

EEG studies yielded similarly mixed findings. Arpaia et al. (2022) reported decreased high-beta power following NF-MED training regardless of the meditation strategy. Participants rated strategy difficulty differently, but ratings were not predictive of neural outcomes. Kosunen et al. (2016) found no significant differences between FA and body scan, yet reanalysis by Salminen et al. (2024) revealed strategy-specific spectral patterns, specifically stronger frontal theta during FA, and gamma during body scan. Salminen et al. (2018, 2022) further showed that dyadic meditation in virtual reality enhanced affective interdependence, with alpha asymmetry correlating with empathy in the EEG-neurofeedback condition. Soriano et al. (2024) demonstrated successful alpha modulation during training, with effects persisting into post-training rest, suggesting partial transfer. Finally, Brandmeyer and Delorme (2020) reported increases in FMT following neurofeedback, though concurrent rises in alpha, beta, and gamma, as well as minimal behavioral change, limit interpretation.

#### 3.7.3 NF-MED in non-clinical adolescents

Three distinct studies (one independent sample) investigated NF-MED and explored associations with self-referential thought and mindfulness-related qualities (Cosgrove et al., 2024; Kirlic et al., 2021, 2022). An active baseline condition where participants judged whether presented adjectives described them, as well as an FA-only condition, was used as controls. In this sample, PCC activity significantly decreased more during NF-MED than controls, but effects were not conserved during the transfer task. While no significant changes were found between DMN regions, strong correlations emerged between PCC and mPFC, dorsal ACC, and posterior INS (pINS), as well as between the DMN and the SN. INS–PCC correlations were only significant during the FA-only run, except for pINS–PCC, which remained significant across two neurofeedback runs. Distinct INS subregions showed differential modulation across conditions, with the pINS demonstrating inverse patterns during neurofeedback versus transfer and FA-only runs. While subjective experiences were linked with feedback, self-reported measures such as ease of focus and psychological measures of well-being and life satisfaction did not differ across conditions or correlate with target regions, respectively. Taken together, findings from novice samples converge in showing that while participants can learn to modulate neural targets with NF-MED, these effects rarely generalize beyond training and translate into behavioral change in an inconsistent manner.

### 3.8 NF-MED in clinical cohorts

Eleven studies (nine independent samples) involved cohorts of individuals specifically selected based on clinical diagnoses. Five studies (four samples) focused on individuals with schizophrenia or schizoaffective disorder (Bauer et al., 2020, 2025; Morfini et al., 2024; Zhang, Bauer, et al., 2025), three studied individuals diagnosed with anxiety and depressive disorders (C. Chen, Xiao, et al., 2021; C. Chen, Yu, et al., 2021; Zhang et al., 2023), and the remaining two investigated overweight/obese participants (Percik et al., 2019) or individuals who experienced early life adversity (Yu et al., 2025). Three of the clinical samples involved adolescent participants. Note that studies do not separate schizophrenia and schizoaffective disorder, thus we report on them together. Anxiety disorders were also not further specified beyond indication of diagnosis by professional psychiatrists.

All schizophrenia-spectrum-related studies used fMRI and noting or insight meditation as the primary intervention to reduce auditory hallucinations by modulating self-referential brain networks. NF-MED group successfully modulated connectivity within the DMN and decreases in connectivity between the DMN and both the CEN and language-related regions (e.g., STG and auditory cortex). Symptom outcomes were mixed, given that Bauer et al. (2020) reported significant between-group differences at 1 week follow-up, while others did not. These studies are limited by the lack of a mindfulness-only control condition, a single neurofeedback session, and assessment of hallucination prevalence and severity only post-neurofeedback rather than at pre- and post-intervention timepoints, despite some of them being among the few RCTs included in the current study sample.

In three studies on anxiety disorders (two samples), NF-MED reduced anxiety and increased state mindfulness. Particularly, Zhang et al. (2023) found reduced connectivity within DMN areas (specifically between PCC and medial prefrontal cortex as well as reduced DMN-CEN connectivity, which correlated with neurofeedback performance and significantly predicted improvement in mindfulness across various dimensions. While C. Chen, Xiao, et al. (2021) found significant group differences, they also found significant power increases across three frequency bands (alpha, theta, and gamma) even though only alpha was the neurofeedback target. Cross-study comparisons were limited by differing samples (adolescents versus adults), modalities (fMRI versus EEG), meditation intervention (noting/insight vs mindfulness), and small sample sizes.

Yu et al. (2025) investigated PCC-targeted fMRI NF-MED in adolescents with early life adversity, comparing them with healthy controls across neurofeedback and sham conditions. PCC activation was downregulated during NF-MED rather than during non-NF-MED conditions, but no significant group or interaction effects emerged. Exploratory analyses showed greater deactivation in healthy controls and stronger PCC–ACC connectivity in the early life adversity group. Positive affect and perceived stress improved for early life adversity participants receiving NF-MED, though effects on mindfulness and negative affect were inconsistent or not sustained. One additional study examined the application of NF-MED in obesity (Percik et al., 2019) and reported improvements at a 3-month follow-up (weight loss, emotional eating, anxiety, and depression), but was limited by small sample size, lack of control groups, and absence of controls for confounding factors such as medication use and replication. Furthermore, while the authors report implementing mindfulness and focus tasks, exact instructions are not reported, complicating understanding of which exact activity the neurofeedback supports. While these findings support the feasibility of NF-MED interventions in these populations, definitive conclusions remain premature.

## 4 Discussion

The intersection of neurofeedback and meditation research remains at an exploratory stage with highly heterogeneous methodology and largely dominated by proof-of-concept feasibility studies (Thibault et al., 2016), as evidenced by a mean sample size of 30 participants. Similar to phase I clinical trials, most studies prioritize proof-of-concept demonstrations and safety over standardized protocols or reproducible outcomes (Sorger et al., 2019). While existing studies provide evidence that neurofeedback can modulate brain activity, such modulation is expected given that this technique is designed to alter brain activity through operant conditioning. Evidence that these changes meaningfully translate to behavioral gains, participant phenomenology, or transferable skills remains inconclusive. This gap and asymmetry are a central constraint and limitations for making translational claims.

While the state-of-the-art may appear reproducible (Thibault et al., 2018), closer inspection reveals widespread heterogeneity. Studies vary in aims, methodology, outcome measures, meditation interventions, neural targets, feedback design, and participant characteristics. For example, Ganesan et al. (2025) and Prestel et al. (2019) claim to investigate novices and long-term meditators, respectively, but their samples overlap extensively in meditation hours. It is critical to note that the classification of “long-term” meditators is imprecise. Categorizing meditation practices with as little as 100 h of cumulative practice as “long-term” may not reflect the development of meditative outcomes or traits, and risks conflating early-stage practitioners with genuinely long-term individuals. A more standardized definition of what constitutes a “long-term” meditator is essential for advancing research in this area. Without clearer criteria, the comparison of neurofeedback outcomes between novices and long-term meditators becomes increasingly unreliable. There is a critical need for a consensus on what qualifies as “long-term,” as this designation often shapes study design and interpretation of results. Progress toward rigorous NF-MED protocols will require clearer phenomenological landmarks of practice (beyond cumulative practice hours), methodological coherence, theoretical clarity, and improved reporting (Sparby & Sacchet, 2022). Such advances are a prerequisite for testing developmental claims, including, in the longer term, claims about advanced meditation.

Phenomenological landmarks may include structured reports of both the neurofeedback intervention and the meditation experience itself. For example, post-session reports could assess how the feedback signal was experienced and used during practice, including the extent to which participants relied on the signal, whether they experimented with different strategies to influence it, and how feedback shaped attentional engagement or mental effort. At the level of the meditation practice, landmarks may include experiential markers such as attentional stability, perceived effort versus effortlessness, frequency of mind-wandering, changes in sensory or interoceptive clarity, and shifts in affective tone (Lutz et al., 2025; Sparby & Sacchet, 2022). In some cases, these reports may also capture state or stage transitions in practice, such as the shift from effortful concentration to more stable attention or broader monitoring of experience. Systematically tracking such phenomenological features would allow neural modulation to be interpreted in relation to identifiable phenomenology of states or stages of meditation rather than signal control alone. These challenges relate to broader issues in contemplative science (Davidson & Kaszniak, 2015), and should, therefore, be addressed to establish an increasingly rigorous science of NF-MED.

Across studies, the most consistent neural finding is the downregulation of the PCC and DMN, typically associated with and interpreted as reduced self-referential processing and increased equanimity (Brewer et al., 2013; Davey et al., 2016; Lord et al., 2025). Yet this apparent convergence is only superficial as “self-reference” is operationalized in diverse and sometimes incompatible ways, and findings vary widely across regions and frequency bands. Furthermore, most studies primarily assess the ability to volitionally modulate PCC/DMN activity during neurofeedback, rather than demonstrating that such modulation reliably tracks behavioral or phenomenological indices of a well-defined meditative process. DMN activity is known to be sensitive to multiple non-specific factors, including attentional effort, task engagement, arousal, and fatigue, raising the possibility that observed downregulation reflects generic cognitive control or task compliance rather than meditation-specific mechanisms (Menon, 2023). Interpretation is further complicated by the functional heterogeneity of the DMN itself, which comprises multiple subsystems involved in distinct processes such as memory, valuation, and internally directed cognition (Menon, 2023). In other words, there is a risk of conflating operant control of neural signals with meditative processes. Such conflation may lead to overinterpretation of PCC/DMN modulation as an index of meditative depth in NF-MED paradigms.

Focused attention practices appear to yield more reliable effects than open-monitoring or noting, but this may reflect sampling biases rather than genuine differences in meditation practice effects, given that considerably more studies employed FA than any other meditation practice, especially in long-term meditators (see Tables 2–4). More fundamentally, NF-MED research is split between two competing framings, meditation as a top–down exercise in attentional control versus meditation as cultivating effortlessness and non-striving. Other dimensions such as interoception, despite robust evidence for its role in mindfulness and its relevance to the sense of self (Engelen et al., 2023; Farb et al., 2015; Gibson, 2019), have not been investigated. These gaps highlight the need for a more systematic and theoretically grounded mapping of neurofeedback targets onto specific dimensions of meditative practice.

### 4.1 Research design and methodological considerations

In the following sections, we highlight design and methodological gaps and propose possible solutions. Briefly, current research designs in NF-MED studies face several recurring limitations: sample size, the lack of comparative studies, poorly defined control conditions, vague reporting on meditation instruction, and the absence of longitudinal follow-up assessments. Only one study directly compared novice and long-term meditators (Van Lutterveld et al., 2017). Moreover, expertise is consistently operationalized by practice hours, but this index is increasingly recognized as insufficient for capturing meditative depth or skill (Ehmann et al., 2024).

#### 4.1.1 Sample size

Given that the relevant question to answer is not whether NF-MED works at all, but whether it provides an improvement over meditation alone (or pharmacological treatments in clinical populations), the adequacy of current sample sizes depends crucially on the level of inference being made. The median sample size of the reviewed studies is *N* = 28, which aligns with broader NF literature, where sample sizes are estimated at a median of 20 subjects for fMRI and fNIRS (Fede et al., 2020; Kohl et al., 2020), and 26 for EEG (Rogala et al., 2016). At this scale, studies may be able to detect medium to large effect sizes in simple square designs but are under-powered for reliably detecting transfer or group effects necessary for translational applicability (Fede et al., 2020; Thibault & Pedder, 2022).

The limitation is, therefore, not that existing studies are “small,” but that their sample sizes are mismatched to the claims often made. While current designs can support mechanistic or feasibility-level inferences, larger and more standardized cohorts are required to support quantitative synthesis (e.g., meta-analysis) and to establish reliable effects across populations and protocols. However, increasing sample size is rarely straightforward in NF-MED research. For instance, recruiting 60 participants for 10 sessions each (e.g., 6 training sessions, 2 transfers, and 2 follow-ups) would require 600 individual acquisition sessions. Given the substantial logistical, scheduling, and financial constraints involved, especially for high cost of technologies such as fMRI, researchers often face a practical trade-off between increasing N and implanting longitudinal designs with sufficient training and follow-up. Furthermore, attrition rates are commonly observed in mindfulness and meditation research (Lam et al., 2022), further compounding this challenge.

An alternative to increased sample sizes is increasing effect sizes by identifying protocol responders a priori. This is common practice in personalized medicine and precision intervention frameworks, which aim to determine which individuals are most likely to benefit from a given protocol before it is administered rather than retrospectively following data collection, thereby enriching samples for probable responders and improving statistical power without inflating Type I error. In the NF-MED case, this could, for example, involve identifying the stage of meditative development at which neurofeedback may most likely boost desired effects, yielding short and targeted interventions (Grabovac et al., 2026). Such stratification could improve detection of protocol-specific effects without proportionally increasing logistical burden. In this sense, integrating theoretically informed responder profiling into study design may be a more realistic and conceptually aligned strategy for advancing NF-MED than relying exclusively on larger, yet often impractical, sample expansions.

#### 4.1.2 Control and baseline conditions

Control conditions remain among the most pressing methodological issues. While neurofeedback outperforms no-feedback conditions, this does not demonstrate that modulation of the intended target drives outcomes. Target specificity remains largely untested (Gruzelier, 2014b), and sham versus active feedback often shows no differences, a concern that is likely underestimated given file drawer scenario and publication bias. Only 6 out of 30 studies included a meditation-only control group to control for non-neurofeedback-related outcomes, leaving open whether observed effects are attributable to neurofeedback or to meditation itself. Equally concerning is the lack of bidirectional or differential feedback designs—that is, designs where participants must modulate neural activity in the opposite direction than the target, or to achieve different levels of modulation (i.e., graded activation). These designs have the potential to mitigate major confounds, for instance by demonstrating neurophysiological specificity and excluding spatial non-specific effects or placebo (see Sorger et al., 2019, for a detailed discussion of control conditions in NF-MED studies). Additionally, no study has yet compared neural feedback effects with those achieved through traditional teacher-guided instruction based on phenomenological inquiry and verbal report. This is a crucial omission given that establishing NF-MED’s advantage over traditional contemplative pedagogy is essential for determining its translational value.

Furthermore, important confounds such as breath and cardiac variability are rarely monitored. Given the prominent focus that many practices accord breathing generally, as well as known meditation-related physiological effects (Sezer & Sacchet, 2025), cardiorespiratory variability is likely to be an important confound requiring well-designed controls. This is especially relevant in fMRI studies, where a known effect of physiological variables modifying blood-dependent oxygenation levels risks conflating cardio-respiratory and neural effects (Thibault et al., 2018).

Resting baseline conditions are also problematic as highly experienced meditators may spontaneously enter meditative states even during rest (Kakumanu et al., 2018; Tang et al., 2012; Yang, Chowdhury, et al., 2024). Without concurrent phenomenological reporting, this control condition may be invalid. Alternative tasks that elicit cognitive effort without meditative phenomenology (e.g., as backward counting or working memory) may offer more suitable baselines (Yang, Chowdhury, et al., 2024). However, these tasks have not yet been validated in novice samples. Objective measures of meditative depth, such as heart-evoked potentials (Nath et al., 2025) or dereification markers (Madl, 2024), could function as complementary markers, but they remain under development. Until these tools mature, future research should prioritize well-defined control tasks, meditation-only comparators, and non-meditative attention or affective tasks.

#### 4.1.3 Longitudinal follow-ups

Unlike clinical neurofeedback protocols that routinely include follow-ups ranging from 1 week to 3 months to assess the durability of learning and symptom change, NF-MED rarely includes follow-ups beyond the training session. This is a crucial gap as meditative insights often unfold gradually, requiring integration over time (Galante et al., 2023; Shi, 2021). Follow-up assessments are necessary to determine whether neurofeedback supports motivation to continue practice or experiential insights over time (Ganesan et al., 2025). Without longitudinal evidence, the developmental impact of NF-MED will remain speculative.

#### 4.1.4 Meditation instruction and teacher involvement

Meditation instructions are often too vague, despite evidence that different meditation styles produce diverse autonomic and neural effects (Brandmeyer et al., 2019; Lumma et al., 2015; Sezer & Sacchet, 2025). This makes it difficult to determine what participants were actually practicing or to compare across studies. Nonetheless, flexible approaches may be more realistic for novices, such as allowing participants to explore and reflect on strategies (Prestel et al., 2019; Thibault et al., 2018). None of the reviewed studies included live instruction from meditation teachers, and only three used recorded instructions, presumably by MBSR instructors. While this is in line with the mindfulness literature (Strohmaier, 2020), it overlooks that novices typically require guidance to navigate meditative experiences, especially when these meditative experiences may be intensified and destabilized by feedback and removed from traditional interpretative contexts. Teachers provide not only instructional precision but also containment and support.

Without proper instruction, it is difficult to assess whether neurofeedback meaningfully enhances meditation practice or simply adds an appealing but unnecessary technological layer. Future research must clarify whether neurofeedback provides unique benefits beyond those achievable through conventional instruction, and if so, at which stage of practice they become relevant and most impactful. Otherwise, NF-MED risks being implemented as a novelty or optimization aid for wellness consumers, rather than as an evidence-based support for genuine contemplative development.

#### 4.1.5 Feedback modality

Nearly all studies relied on visual feedback, with only a handful using auditory or combined modalities. Yet many meditation practices are performed with eyes closed, raising the possibility that visual feedback disrupts meditative depth or adds cognitive and sensory load. Although virtual-reality-based feedback has been explored to increase immersion (Cohen et al., 2016), whether such environments facilitate or hinder the meditative process remains unclear. Requiring participants to open their eyes periodically to view feedback (as in Van Lutterveld et al., 2017) may also introduce signal artifacts, though no differences were reported between eyes-closed and eyes-open conditions in that study. Relatedly, four studies reported effects in the gamma frequency range. However, studies using gamma-band activity as a feedback target should exercise caution, as gamma signals can be contaminated by muscle activity (Whitham et al., 2007). Finally, despite some early comparisons (Ejaz et al., 2024; Hinterberger et al., 2004; Ishihara et al., 2020; Weiskopf, 2012), strong evidence regarding optimal feedback modalities remains lacking, and is likely to depend on individual learning preferences and the nature of the meditative task.

#### 4.1.6 Outcome measures and participant engagement

Defining “success” in NF-MED remains another challenge (Davidson & Kaszniak, 2015; Paret et al., 2019). Neurofeedback typically emphasizes signal modulation, while meditation studies emphasize stress reduction, attention, or mindfulness, and often via self-report. Yet these scales may capture only distant correlates of practice and often fail to capture phenomenological change. Studies reporting discrepancies between neural and behavioral or experiential outcomes are common as well. More precise mappings between neural patterns and meditative states are needed to ensure that neurofeedback targets correspond to meaningful experiential changes. While two studies (Kosunen et al., 2016; Salminen et al., 2024) indicate that virtual-reality-based meditation, with or without neurofeedback, was associated with fewer negative affective experiences such as boredom or restlessness in novices, few studies probe participants’ actual engagement such as strategies, adherence to instructions, or adaptation in response to feedback, yet such reports are critical for building personalized, meaningful closed-loop neurofeedback systems that support long-term meditative development. For instance, brief post-session self-reports at the end of each session could be combined with neural and physiological data to train machine learning algorithms that tailor neurofeedback parameters (e.g., duration, difficulty, practice type, attention, sensations, mind-wandering) to the individual.

### 4.2 Recommendations for the future of the field

Despite encouraging proof-of-concept studies, NF-MED research remains heterogeneous, making it difficult to support definitive claims about its application. Future research must address several foundational questions if NF-MED were to be used as a clinical intervention in the future.

#### 4.2.1 Clarify the functional role of neurofeedback in meditation learning

A priority is to determine whether neurofeedback can actively accelerate meditative learning, supporting the development of specific meditative skills, and helping practitioners overcome meditative barriers such as anxiety, fear, or self-doubt (Yang, Sparby, et al., 2024). Key outcomes could include whether neurofeedback improves daily practice adherence, strengthening discrete meditative capacities (e.g., the ACAM-J factors in concentrative meditation), and supports advanced meditation practice. To date, most studies have focused on whether meditation enhances neurofeedback performance; there is a parallel need to investigate whether neurofeedback improves meditation adherence, learning, and transfer. Approaches such as those used in Dziego et al. (2024), which link mindfulness adherence to neurofeedback outcomes, could be adapted to assess whether neurofeedback involvement predicts sustained meditation engagement. More broadly, neurofeedback offers an underexplored opportunity to probe the neuroplastic mechanisms of meditation, including how effects vary across early, intermediate, and advanced stages of practice, and whether NF-MED promotes flourishing beyond what is achievable with other contemplative or cognitive training methods.

#### 4.2.2 Standardize study designs while embracing individual precision

NF-MED research progress would require greater homogeneity in methodological development such as standardized outcome measures, reporting practices, and study protocols to enable direct comparison and meta-analytic synthesis. Concurrently, meditation and neurofeedback are inherently individualized processes where practitioners vary in baseline neural dynamics, meditative traits, and proficiency. Thus, precision should be increased where individual differences matter (Brandmeyer et al., 2023). This could be applied to, for example, tailored-EEG feedback to individualized alpha frequency (Smulders et al., 2018) or subject-specific regions of interest (ROIs) in fMRI-based neurofeedback. Increasingly systematized training frameworks wherein variables can be methodically manipulated to elicit predictable outcomes would open the space for meaningful individualization. This may be achieved by borrowing principles from sports science and elite performance training that combine objective training predictors with the trial-and-error process shaped by the athlete’s situated context (as proposed in Ehmann et al., 2025). Thus, NF-MED could adopt continuous performance monitoring, adaptive feedback, and progressive skill building, with machine learning algorithms optimizing training duration, intensity, density, and content, balancing accelerated learning with long-term retention, and minimizing adverse effects. This approach could lay the groundwork for a mature science of NF-supported meditative development and will be essential for developing NF-MED into a reproducible, mechanistically grounded, and individually responsive framework capable of supporting both scientific understanding and transformative contemplative training.

Choice of neuroimaging modality will also fundamentally shape the future accessibility and scalability of NF-MED. As discussed in the context of sample size constraints, high acquisition burden directly limits feasible sample size and longitudinal data collection. fMRI offers high spatial resolution and more direct access to deep cortical and subcortical targets, but its high financial cost, immobility, and infrastructure demands limit sample sizes and ecological validity. The acoustic and physical constraints of the MRI system environment may further interfere with meditative depth, potentially limiting inclusion to only particularly advanced practitioners—who are likely to benefit the least from such technologies. EEG, by contrast, provides high temporal resolution and portability at comparatively low cost, enabling repeated and potentially home-based training, though at the expense of spatial specificity, susceptibility to artifact, and possible participant discomfort. While EEG thus lends itself well to consumer-grade neurofeedback, a recent meta-analysis found no evidence that consumer-grade devices allow participants to modulate their brains and experience psycho-behavioral benefits (Treves et al., 2025). fNIRS occupies an intermediate position in cost and spatial resolution but remains constrained in cortical depth coverage, and remains under-tested in NF contexts (Kohl et al., 2020).

While the present review intentionally focused on neural targets, non-neural modalities such as heart rate variability or electrodermal activity, may also provide meaningful regulatory targets for NF-MED. These specific markers could be closely linked to meditative stage (Cain, Brandmeyer, et al., 2024; Sezer & Sacchet, 2025) and alter in specific ways during different meditative practices (Lumma et al., 2015; Soriano, Rodriguez-Larios, et al., 2024). Unlike neural signals, they are inexpensive, portable, and less technically demanding, which may enhance accessibility and scalability. Biofeedback may be especially interesting given that practitioners are often instructed to be aware of passing sensations (Anālayo, 2021; Burbea, 2014; Ingram, 2020; Sayadaw, 1994), including the breath (Sekida, 2005) and heart energy (Rinpoché, 2022), and may thus function as a magnifying glass. In the future, review of concurrent biofeedback and meditation may indicate whether peripheral feedback can meaningfully support meditative development while highlighting modality-specific methodological issues.

#### 4.2.3 Specify primary and secondary outcomes

Future NF-MED studies should more clearly state the primary and secondary outcome measures. Similar to randomized controlled trials, stating clearly defined outcomes allow for focused hypothesis testing, exploratory analyses, and better study design and reproducibility. This distinction should apply not only to behavioral and phenomenological outcomes but also to neural outcomes. At present, studies treat the ability to modulate neural signals during training as evidence of success. However, signal modulation alone should be considered an intermediate mechanistic outcome rather than a final endpoint, as meaningful effects should ideally translate to changes in behavior, meditative skills, or phenomenological experience. Future research should, therefore, explicitly define what constitutes meaningful transfer beyond signal modulation, such as persistence of neural effects outside the feedback context or improvements in relevant behavioral or experiential measures. Outcome selection should also be justified relative to hypothesized mechanisms linking neural targets to meditative processes. Relatedly, preregistration of hypotheses, outcome measures, and analytic strategies would help reduce interpretive flexibility and improve transparency and comparability across NF-MED studies.

#### 4.2.4 Develop scalable closed-loop NF systems that integrate ethical and existential safeguards

Research in meditation may seek to harness neurofeedback to enable personalization of meditative development with the same precision seen in high-performance athletic or skill training, optimizing both the pace and quality of learning. Closed-loop neurofeedback systems could enable real-time adaptation to a practitioner’s current meditative stage and ongoing state fluctuations by modeling the practitioner’s expected trajectory and adjusting feedback targets dynamically to minimize prediction error relative to desired meditative states. This could, in theory, include training that focuses on specific obstacles or “hindrances” (e.g., restlessness, drowsiness) as they arise, or even anticipating adverse effects to aid navigating these safely, for example, by switching to lower practice doses or to grounding practices (Ehmann et al., 2025).

Based on phenomenological landmarks—such as those described at the beginning of our discussion section—and the neural–physiological correlates associated with them, the system would provide real-time adaptation of neural target, modulation intensity, and type of practice. This may be analogous to a meditation teacher pointing out obstacles to deeper progression, with additional validated markers and correlates not accessible in a traditional setting. Given the potential for accessibility and scalability of such devices down the line (given the relatively lower cost of EEG technology), they hold the potential for supporting the user beyond teacher’s capacity, in daily life or between teacher-guided meditation sessions.

Moreover, research-grade wearable and semi-portable neurotechnology offers a scalability strategy that could address several constraints driving the current small-sample, feasibility-focused NF-MED literature, without endorsing consumer devices. These technologies reduce per-session setup burden and increase scheduling flexibility. Thus, scalable acquisition architectures could support larger samples and more repeated sessions, improving statistical power and sensitivity to small effects while reducing attrition and participant fatigue in longitudinal designs due to preparation and set up time needed for fMRI or EEG. These platforms may also enable more ecologically valid measurement by capturing practice in naturalistic contexts and across days or weeks, rather than relying on a small number of laboratory sessions.

However, it remains to be seen whether, and in which situations, such technologies provide an advantage over traditional settings—where the inter-personal space, teacher’s experiences and expertise, as well as direct transmission, may provide invaluable dimensions to practice beyond what technological devices can offer (a topic that is itself under-researched and under debate, see Brown et al. (2021) for a discussion of similar questions in the field of psychiatry and AI; Canby et al. (2024) for a discussion on teacher impact on meditation learning in Buddhist meditators). Similarly, despite the promise, accelerating meditation development through technological means raises questions about the preservation of the spiritual, ethical, and existential dimensions of contemplative practice (Elf et al., 2023). Drawing on recent work on contemplative hindrances and readiness to react if such accelerations were to happen (Sparby & Sacchet, 2022, 2025b), future NF-MED protocols should incorporate safeguards to prevent destabilizing effects from premature or accelerated meditative advancement. Specifically, these safeguards should include (1) readiness assessments to evaluate participant preparedness for intensive practice; (2) staged progression criteria informed by traditional meditation maps; (3) active monitoring and availability of mental health professionals or experienced meditation teachers qualified to address negative psychological reactions and distress, following standard practices in medical and psychological research; (4) integration of ethical and character development alongside technical skill acquisition, as traditional contemplative frameworks emphasize moral virtues as foundations for safely navigating potent meditative insights; and (5) equitable access considerations to prevent NF-MED technologies from becoming exclusive commodities available only to privileged populations. The field must ensure that NF-MED augments practice responsibly, without reducing meditation to a mechanistic optimization tool divorced from its broader cultural and ethical context.

## 5 Conclusion

NF-MED research is still in its infancy, and its promise toward the possibility of supporting advanced meditation has yet to be fully tested and realized. Toward this potential, this research domain must overcome several challenges: methodological heterogeneity, insufficiently defined meditation instructions, poorly designed or absent control conditions, ambiguous outcome measures, and the lack of longitudinal follow-up. To advance the field, future work must adopt more homogeneous study designs, incorporation of more meaningful control conditions, and clearer reporting of meditation practice. Equally important is the inclusion of meaningful behavioral and phenomenological outcome measures. If these challenges are met, NF-MED may become an innovative framework that bridges neuroscience, technology, and contemplative traditions, supporting clinical applications, and opening new avenues for the science and practice of human flourishing.

## Data Availability

As this article is a systematic review and does not involve the collection or analysis of original data, no datasets or code are associated with this work.
